# Supplementary material for: Immunomodulatory Drugs Alter the Metabolism and the Extracellular Release of Soluble Mediators by Normal Monocytes
Source: Molecules. 2020 Jan 16;25(2):367. doi: 10.3390/molecules25020367 (PMC7024334; doi:10.3390/molecules25020367)
Supplement: Supplementary file 1 [file molecules-25-00367-s001.pdf]

SUPPLEMENTARY INFORMATION

IMMUNOMODULATORY IMID DRUGS ALTER THE METABOLISM AND THE EXTRACELLULAR RELEASE OF SOLUBLE MEDIATORS BY NORMAL MONOCYTES

Ida Marie Rundgren[1, 2], Anita Ryningen[1], Tor Henrik Anderson Tvedt[3], Øystein Bruserud[2, 3], Elisabeth Ersvær[1]

[1]Department of Biomedical Laboratory Scientist Education and Chemical Engineering, Faculty of Engineering and Natural Sciences, Western Norway University of Applied Sciences, Bergen, Norway; [2]Department of Clinical Science, University of Bergen, Bergen, Norway and [3]Section for Hematology, Department of Medicine, Haukeland University Hospital, Bergen, Norway.

Supplementary Table 1. The spontaneous release of soluble mediators by normal monocytes derived from fifteen healthy individuals; an overview. The data are presented as the median and range of the levels detected in culture supernatants. All concentrations are given as pg/ml.

Mediator; classification of each mediator	Spontaneous release
**Chemokines**	
CCL1	7,5 (<1.3-633)
CCL2	405 (4,7-16,374)
CCL3	768 (389-62,216)
CCL4	752 (269-21674)
CCL7	151 (8.0-1636)
CXCL1	493 (60.7-18,669)
CXCL10	11,5 (0.1-100)
**Interleukins**	
IL1β	14,1 (1.2-1116)
IL1RA	2462 (50.1-20,635)
IL6	37.3 (0,47-1645)
IL8/CXCL8	29 (0.6-1371)
IL10	11.2 (<1.9-402)
**Other mediators**	
MMP9	8193 (17.6-21,706)
TNFα	23 (0.6-1371)

Supplementary Table 2. The effects of thalidomide, lenalidomide and pomalidomide on TLR4/LPS induced release of soluble mediators by normal monocytes derived from healthy individuals; a comparison of the effects of the three pharmacological agents. The data are presented as the uncorrected p-values for each of the statistical comparisons, and the drug with the highest level is given together with the corresponding p-value for statistically significant differences in the paired comparisons. Fifteen healthy individuals were examined. The Wilcoxon's test for paired samples was used for the statistical analyses (ns, not significant).

Mediator; classification of each mediator	Thalidomide versus lenalidomide	Thalidomide versus pomalidomide	Lenalidomide versus pomalidomide
**Chemokines**			
CCL1	ns	ns	ns
CCL2	0.001 Lenalidomide	0.001 Pomalidomide	0.002 Pomalidomide
CCL3	ns	0.005 pomalidomide	0.009 Pomalidomide
CCL4	0.001 Lenalidomide	0.001 Pomalidomide	0.001 Pomalidomide
CCL7	0.004 Lenalidomide	0.001 Pomalidomide	0.001 Pomalidomide
CXCL1	ns	ns	ns
CXCL8	0.015 Lenalidomide	0.009 Pomalidomide	0.041Pomalidomide
CXCL10	ns	ns	ns
**Interleukins**			
IL1β	0.001 Lenalidomide	0.001 Pomalidomide	0.001 Pomalidomide
IL1RA	ns	ns	ns
IL6	0.041	0.003 Pomalidomide	0.001Pomalidomide
IL10	ns	ns	ns
**Other mediators**			
MMP9	0.003 Thalidomide	0.001 Thalidomide	0.003 Lenalidomide
TNFα	0.001Thalidomide	0.031 Pomalidomide	ns

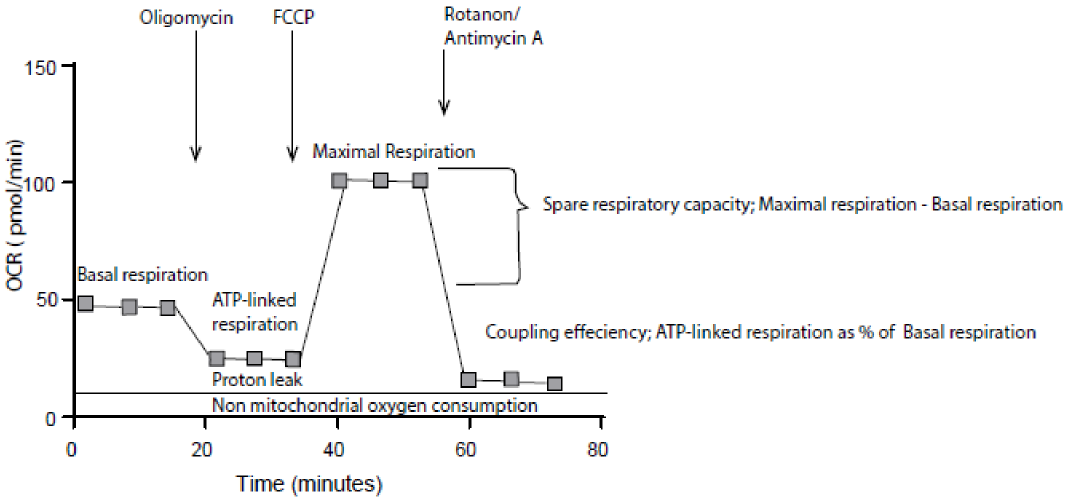

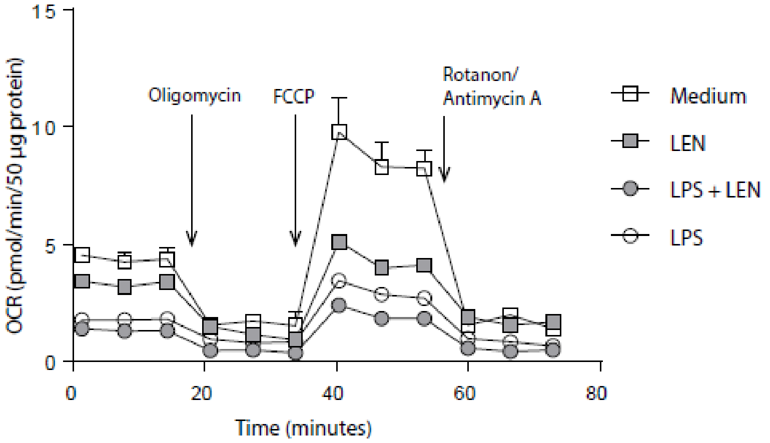

Supplementary Figure 1. Analysis of monocyte metabolism using The XF Mito Stress Test assay and the Seahorse XF 96 cell analyzer; an overview of the Seahorse assay (upper part) and the results from a representative experiment (lower part). Normal monocytes were cultured for 4 hours either in assay medium alone, LPS 1 ng/ml, lenalidomide 5 µg/ml or LPS plus lenalidomide before they were analyzed using the XF Mito Stress Test assay. The oxygen consumption rate (OCR) was measured three times during each of the following four periods each of 20 minutes duration; (i) the basal period when cells were incubated in the assay medium alone (the first 20 minutes); (ii) after addition of oligomycin (20-40 minutes); (iii) after addition of FFCP (maximal respiration, 40-60 minutes); and (iv) after addition of rotanol and antimycine A (after 60 minutes. The OCR was measured three times during each period. The results for each time point are presented as the median of six to eight parallel cultures. For the clarity of the presentation in the figure the standard deviations are presented only for the upper LPS+lenalidomide and lower LPS curve; these variations are representative for the variations between parallel cultures in the experiments.

	Medium vs. LPS	Thalidomide	Lenalidomide	Pomalidomide
**CCL1**	0.0884	0.3003	0.0186	0.0071
**CCL2**	0.0125	0.0356	0.0026	0.0012
**CCL3**	0,0010	0.4603	0.0609	0.0031
**CCL4**	0.0015	0.7764	0.0309	0.0026
**CCL7**	0.0063	0.1771	0.0110	0.0029
**CXCL1**	0.0012	0.9096	0.3635	0.1914
**CXCL10**	0.6002	0.3967	0.1401	0.0186
**IL1 β**	0.0007	0.2805	0.0199	0.0012
**IL-1RA**	0.0171	0.3942	0.9096	0.4955
**IL-6**	0.0007	0.0125	0.0007	0.0007
**IL8/CXCL8**	0,0007	0.3066	0.5701	0.2560
**IL10**	0.0077	0.0357	0.0499	0.0745
**MMP9**	0.0052	0.1556	0.9096	0.1556
**TNFα**	0.0007	0.7764	0.0018	0.0268

Supplementary Figure 2. The effects of TLR4/LPS and IMiDs the release of soluble mediators by normal monocytes; a summary of the results for 15 healthy blood donors presented in Table 1 in the article. The Wilcoxon's test for paired samples was used to compare the results for cultures prepared (i) in medium with and without LPS; and (ii) medium with LPS alone versus medium with LPS together with an IMiD (i.e. thalidomide, lenalidomide or pomalidomide). The p-values are indicated for each comparison and mediator. Statistically significant differences are marked with grey.

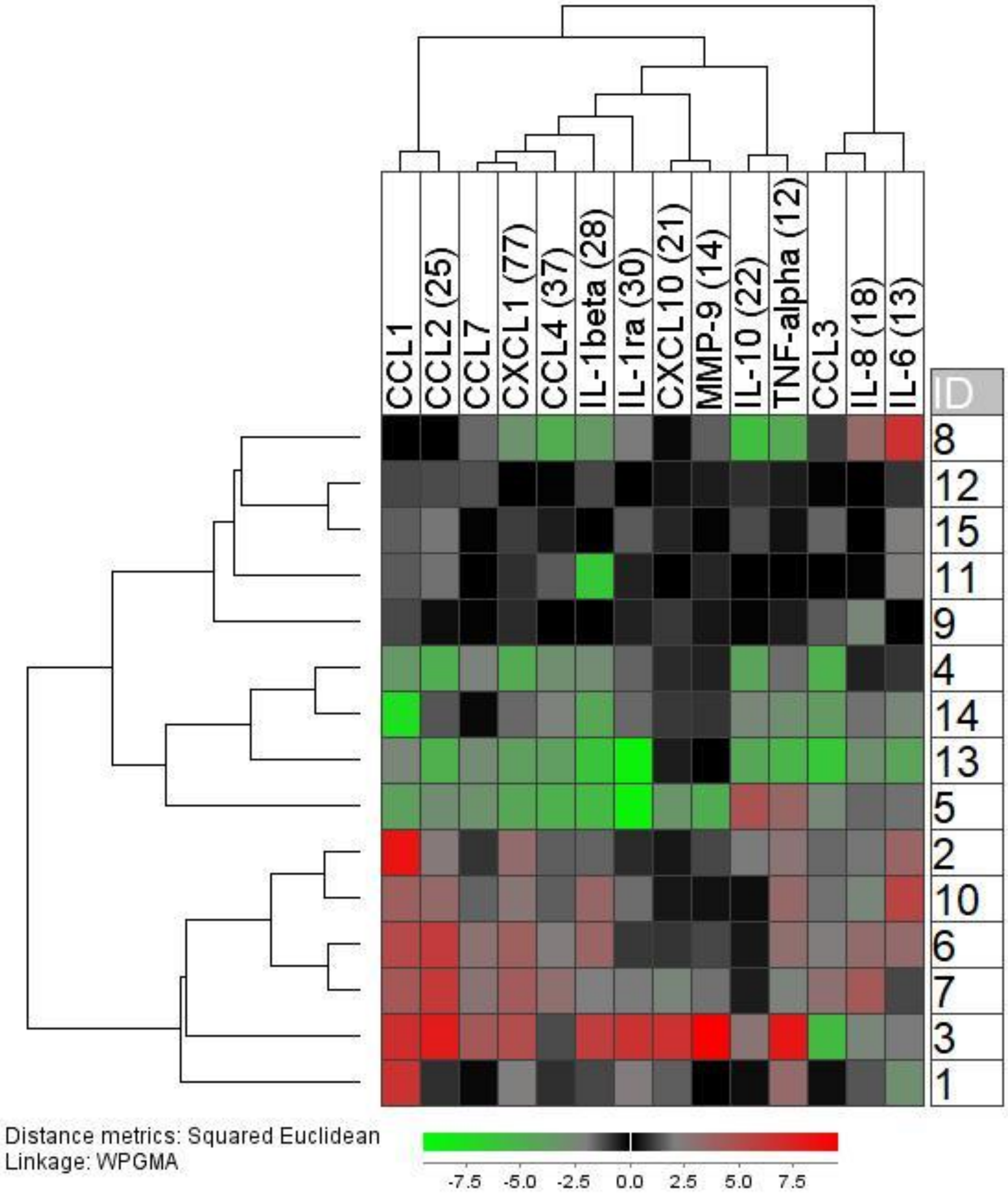

Supplementary Figure 3. A hierarchical clustering analysis of the TLR4/LPS induced stimulation of soluble mediator release by normal monocytes. The analysis was based on the relative responses that were defined as the mediator level in LPS containing cultures relative to the mediator level in control cultures prepared in medium alone. The relative responses were normalized to the corresponding median response before the clustering analysis. The responses varied between individuals and also between mediators, and a lower cluster including six individuals (individuals 1, 2, 3, 6, 7 and 10) showed the strongest TLR4/LPS responses.

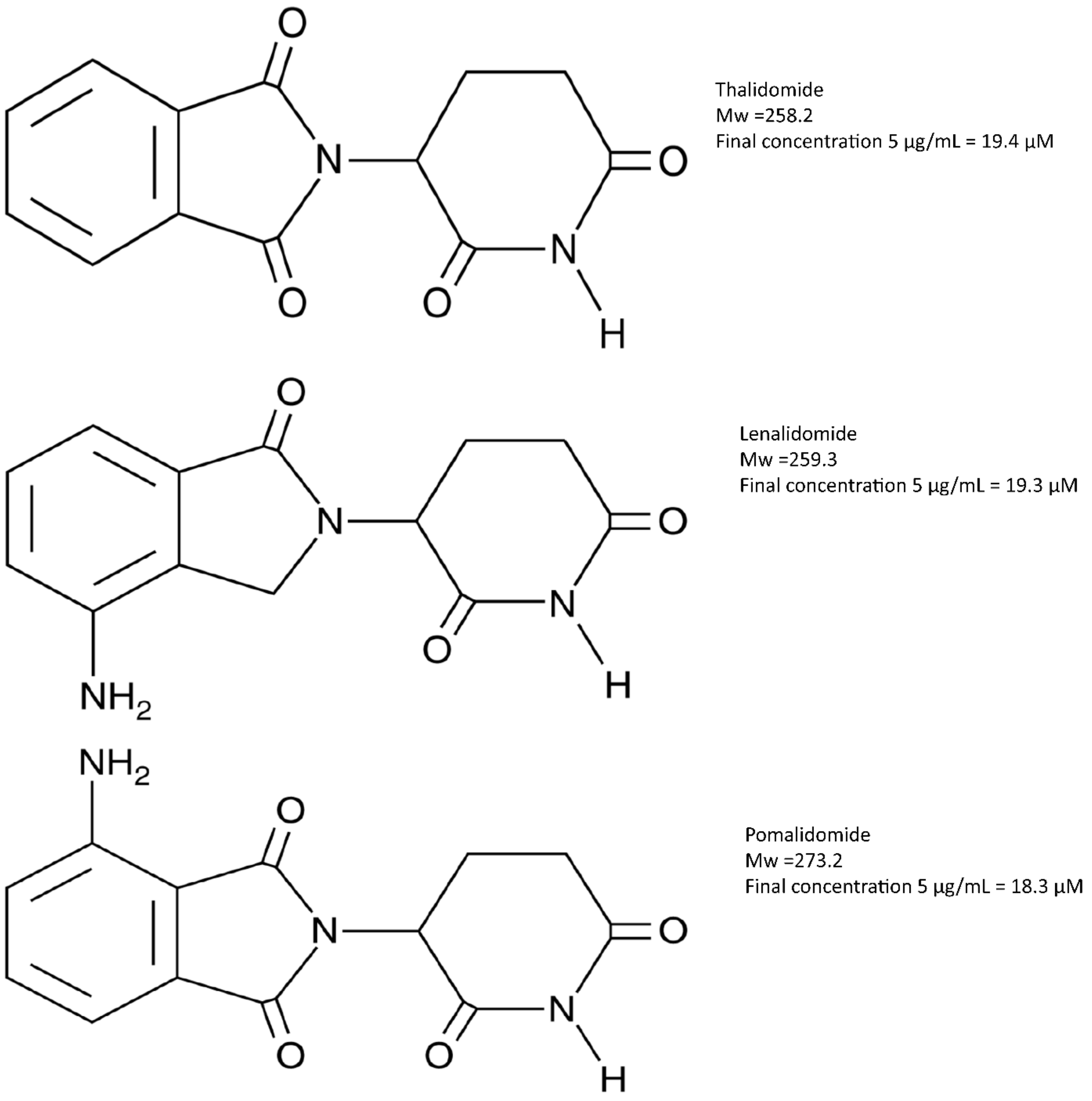

Supplementary Figure 4. The chemical structure of the three IMiDs investigated (thalidomide, lenalidomide and pomalidomide), their molecular weights and the molar concentrations corresponding to 5 µg/mL.
